# Peripheral T Cell Depletion by FTY720 Exacerbates Hypoxic-Ischemic Brain Injury in Neonatal Mice

**DOI:** 10.3389/fimmu.2018.01696

**Published:** 2018-08-06

**Authors:** Josephine Herz, Christian Köster, Marius Crasmöller, Hanna Abberger, Wiebke Hansen, Ursula Felderhoff-Müser, Ivo Bendix

**Affiliations:** ^1^Department of Pediatrics 1, Neonatology and Experimental Perinatal Neuroscience, University Hospital Essen, University of Duisburg-Essen, Essen, Germany; ^2^Institute of Medical Microbiology, University Hospital Essen, University of Duisburg-Essen, Essen, Germany

**Keywords:** neonatal hypoxia ischemia, birth asphyxia, brain injury, T cells, FTY720, immune cell infiltration, neuroinflammation

## Abstract

Hypoxic-ischemic injury to the developing brain remains a major cause of significant long-term morbidity and mortality. Emerging evidence from neonatal brain injury models suggests a detrimental role for peripheral lymphocytes. The immunomodulatory substance FTY720, a sphingosine-1-phosphate receptor agonist, was shown to reduce adult ischemia-induced neurodegeneration through its lymphopenic mode of action. In the present study, we hypothesized that FTY720 promotes neuroprotection by reducing peripheral lymphocytes and their infiltration into the injured neonatal brain. Term-born equivalent postnatal day 9 C57BL/6 mice were exposed to hypoxia ischemia (HI) followed by a single injection of 1 mg/kg FTY720 or vehicle (0.9% sodium chloride). Brain injury, microglia, and endothelial activation were assessed 7 days post HI using histology and western blot. Peripheral and cerebral leukocyte subsets were analyzed by multichannel flow cytometry. Whether FTY720s’ effects could be attributed to its lymphopenic mode of action was determined in T cell-depleted mice. In contrast to our hypothesis, FTY720 exacerbated HI-induced neuropathology including loss of gray and white matter structures. While microglia and endothelial activation remained unchanged, FTY720 induced a strong and sustained depletion of peripheral T cells resulting in significantly reduced cerebral infiltration of CD4 T cells. CD4 T cell subset analysis revealed that circulating regulatory and effector T cells counts were similarly decreased after FTY720 treatment. However, since neonatal HI *per se* induces a selective infiltration of Foxp3 positive regulatory T cells compared to Foxp3 negative effector T cells effects of FTY720 on cerebral regulatory T cell infiltration were more pronounced than on effector T cells. Reductions in T lymphocytes, and particularly regulatory T cells coincided with an increased infiltration of innate immune cells, mainly neutrophils and inflammatory macrophages. Importantly anti-CD3-mediated T cell depletion resulted in a similar exacerbation of brain injury, which was not further enhanced by an additional FTY720 treatment. In summary, peripheral T cell depletion by FTY720 resulted in increased infiltration of innate immune cells concomitant to reduced T cell infiltration and exacerbation HI-induced brain injury. This study indicates that neonatal T cells may promote endogenous neuroprotection in the term-born equivalent hypoxic-ischemic brain potentially providing new opportunities for therapeutic intervention.

## Introduction

Perinatal asphyxia, resulting in hypoxic-ischemic encephalopathy (HIE), is one of the worldwide leading causes of death and disability in term-born children. In high-income countries, 1–6 per 1,000 newborns suffer from HIE during the perinatal period often resulting in cerebral palsy, epilepsy, visual impairment, and motor-cognitive deficits in later life (1). The only clinically approved therapy is hypothermia, which is, however, only effective in 40–50% of patients and has to be initiated in a very short time window (2).

The development of additional or alternative causative therapies that prevent neuronal damage and promote neurological recovery is highly warranted (3). A particularly promising therapeutic target in neonatal hypoxia ischemia (HI) is the post-hypoxic inflammatory response, which involves a variety of innate and adaptive immune cells migrating across the activated blood–brain barrier to invade the brain parenchyma (4–6). The suggested dynamics of leukocyte infiltration into the injured neonatal brain with a peak of T cell infiltration observed at 1 and 7 days and persistence up to 3 months after injury (4, 6, 7) make them amenable to therapeutic intervention.

While the significance of peripheral immune cells for the development of secondary lesion growth in adults has been well established (8, 9), little is known about the functional relevance of different immune cell subtypes in neonatal brain injury. Splenectomy prior to HI in neonatal rats resulted in significant neuroprotection (10). However, the role of distinct immune cell subsets was not addressed. A more specific approach was performed in a very recent work by the use of lymphocyte deficient Rag1^−/−^ mice that revealed significantly reduced HI brain injury compared to wild-type control mice (11). While the selective contribution of T or B cells to the development of brain injury remained uncertain in the latter study, Albertsson et al. specifically focused on gamma delta T cells demonstrating that depletion of this T cell subset provides neuroprotection in postnatal day 4 mice (12). Taken together, currently available data suggest that interfering with cerebral infiltration of peripheral lymphocytes after neonatal HI is beneficial, a hypothesis until now only tested in pre-term and/or inflammation-induced brain injury models (11–13). Studies in term-born equivalent models focusing on hypoxic-ischemic injury are lacking.

FTY720 is an immunomodulatory sphingosin-1-phosphate analog, approved for the treatment of relapsing–remitting multiple sclerosis (14). A major effector mechanism of FTY720 is reduction of peripheral lymphocytes by blocking egress of lymphocytes from lymphoid organs through agonist-induced receptor internalization leading to reduced lymphocyte counts in the injured brain (15). Despite the huge amount of studies reporting neuroprotection in adult brain ischemia (16–20), only two studies focused on potential protection by FTY720 in perinatal brain injury. Previous own work in a pre-term model of oxygen-induced toxicity revealed protective effects that were directly attributed to protection of oligodendrocyte precursor cells and rather independent of FTY720s’ lymphopenic mode of action (21), Yang et al. showed that FTY720 reduces the amount IL-17 producing CD4 T cells in a pre-term model of inflammation-sensitized hypoxic-ischemic brain injury (22). However, the exact mechanisms underlying the protective effects of FTY720 were only partially characterized, e.g., the detailed composition of the CNS immune cell infiltrate and of circulating leukocytes following FTY720 treatment remain unclear.

In the present study, we hypothesized that FTY720 promotes neuroprotection by reducing peripheral T cells and thus infiltration into the injured brain thereby reducing secondary HI-induced brain injury in term-born equivalent mice.

## Materials and Methods

### Animal Care and Group Allocation

Experiments were performed in accordance to the Animal Research: Reporting of *In Vivo* Experiments guidelines with government approval by the State Agency for Nature, Environment and Consumer Protection North Rhine-Westphalia. C57BL/6J mice were bred in house and kept under a 12-h light/dark cycle with food and water *ad libitum*. Bodyweight of pups was recorded at postnatal day 9 (P9), P10, P11, P12, and P16. A total of 275 P9 pups (*n* = 140 female and *n* = 135 male) derived from 40 litters were enrolled. Fifty-seven naïve mice (29 female, 28 male) were used to determine lymphocyte populations in the peripheral blood 1, 3, and 7 days after a single FTY720 injection. Fourteen mice (6 female, 8 male) underwent sham surgery. 12 out of 204 animals (6%) exposed to hypoxia-ischemia died during hypoxia. Remaining animals were randomly assigned to two (saline and FTY720) or four (isotype/saline, isotype/FTY720, anti-CD3/saline, and anti-CD3/FTY720) treatment groups by an independent scientist not involved in data acquisition: the first set of HI mice (*n* = 32; 16 female, 16 male) was used to evaluate brain injury and inflammatory responses *via* histology and western blot 1 week after HI. The second cohort of mice (*n* = 110; 57 female, 53 male) was used for quantification of immune cell subtypes *via* flow cytometry. A third set of mice (*n* = 50, 26 female, 24 male) was used to assess the impact of antibody- and FTY720-mediated T cell depletion and of the combined treatment on HI-induced brain injury *via* histology. In total, two saline and four FTY720-treated mice died between 24 h and 7 days after HI.

### Neonatal Hypoxia-Ischemia

Hypoxic-ischemic (HI) brain injury was induced as previously described (23, 24). Briefly, the right common carotid artery was occluded through cauterization (high temperature cauter, 1,200°C, Bovie, USA) under isoflurane anesthesia (1.5–4 Vol%, total duration of surgery: 5–7 min) followed by 1 h hypoxia (10% O_2_) in an air-tight oxygen chamber (OxyCycler, Biospherix, USA) after 1 h recovery with their dams. Animals were placed on a warming mat (Harvard Apparatus, USA) to maintain nesting temperature during hypoxia (23). Sham-operated were subjected to anesthesia and neck incision only.

### FTY720 Treatment and Antibody-Mediated T Cell Depletion

FTY720 (1 mg/kg body weight, Sigma, #SML 0700 dissolved in 0.9% NaCl) was administered intraperitoneally (i.p.) within 20 min after hypoxia. Dose and administration time point was chosen based on previous studies and experimental reports in adult and neonatal brain injury (19–22, 25). An equal volume of 0.9% NaCl (later referred to “saline”) served as control. Antibody-mediated T cell depletion was performed according to our previous protocol by i.p. injection of 16 µg/g body weight anti-mouse CD3 (Clone 17 A2, BioXcell, USA) every 48 h (26). To determine whether effects of FTY720 were specifically dependent on T cells, antibody depletion was started 24 h prior to HI and prolonged to the end of the experiment. Control mice received 16 µg/g body weight isotype control antibody (Clone LTF-2, BioXcell) at the same time points.

### Tissue Preparation, Histology, and Immunohistochemistry

One week after HI, mice were deeply anesthetized with chloralhydrate (200 mg/kg body weight) and transcardially perfused with ice-cold phosphate buffered saline (PBS). Brains were removed and snap frozen on dry ice. Tissue injury was assessed and scored on cresyl violet stained 20 µm cryostat sections as previously described (23, 27). Briefly, eight regions were scored: the anterior, middle, and posterior cortex, CA1, CA2, CA3, and dentate gyrus of the hippocampus and the striatum. Each region was given a rating from 0 to 3 (0—no detectable cell loss, 1—small focal areas of neuronal cell loss, 2—columnar damage in the cortex or moderate to severe cell loss in the other regions, 3—cystic infarction and gliosis). The sum score from different regions was calculated for each animal resulting in a total maximum score of 24.

Brain tissue loss was determined by measurement of intact areas in ipsilateral and contralateral hemispheres in two sections from the striatal (+0.2 to +0.3 mm from bregma) and two sections from the hippocampal (−1.9 to −2.0 mm from bregma) level using Image J software (NIH, USA). Tissue loss was determined by comparison with contralateral values according to the following equation: [100 − ratio (ipsilateral/contralateral) × 100].

For qualitative assessment of leukocyte infiltration, cryostat sections were stained for the pan-leukocyte marker CD45 as previously described (24). Briefly, tissue sections were thawed and dried at 37°C followed by fixation in ice-cold aceton/methanol (1:1 v/v%) for 10 min at 4°C. Unspecific antibody binding was blocked by incubation with 5% normal goat serum (NGS), 2% BSA, 0.2% Tween 20 in PBS for 1 h at room temperature. Sections were incubated with rat anti-mouse CD45 (1:20, BD Pharmingen, Germany) in 2% NGS, 1% BSA, 0.2% Tween 20 in PBS at 4°C overnight. Antibody binding was visualized by incubation with anti-rabbit Alexa Fluor 488 (1:500, Invitrogen, Germany) for 2 h at room temperature. Nuclei were counterstained with 4′,6-Diamidin-2-phenylindol (Dapi, 100 ng/ml; Molecular Probes, USA). Images were acquired with confocal microscopy (A1plus, Eclipse Ti, with NIS Elements AR software, Nikon, Germany). Confocal z-stacks of 18 µm thickness (z-plane distance 2 µm) large-scale images (stitching) of complete hemispheres were acquired with a 10× objective.

### Western Blot

For western blot analysis 200 µm sections of the ipsilateral hemisphere within the range of −2.0 to −2.3 mm from bregma were dissected and homogenized in ice-cold lysis buffer (RIPA, Sigma-Aldrich) containing protease and phosphatase inhibitors (cOmplete, Roche) and 100 mM PMSF (Sigma-Aldrich). The supernatant was collected and processed as previously described (23, 24). Briefly, after determination of protein concentration using the Pierce bicinchoninic acid assay protein assay kit (Thermo Scientific, USA), protein lysates were separated on gradient sodiumdodecylsulphate (SDS) polyacrylamide gels (Mini-PROTEAN TGX Precast Gels, Any kDa, Bio-Rad, Germany) or 12.5% SDS gels and transferred to nitrocellulose membranes (0.2 µm, Amersham, USA) at 4°C overnight. Equal loading of 20 µg/lane and transfer of proteins was confirmed by staining of membranes with Ponceau S solution (Sigma-Aldrich). Nonspecific binding was blocked by incubation in 5% non-fat milk powder, 0.1% Tween in TBS (TBST). Membranes from gradient SDS gels were incubated with the following primary antibodies: rabbit anti-myelin basic protein (MBP, 1:10.000, Covance, USA), mouse anti-microtubuli associated protein-2 (MAP-2, 1:1,000, Sigma-Aldrich), and goat anti-vascular cell adhesion molecule-1 (VCAM-1, 1:10,000, R&D Systems, USA). Membranes of 12.5% SDS gels were incubated with rabbit anti-ionized calcium binding adaptor molecule-1 (Iba-1, 1:1,000, Wako, Japan) and biotinylated goat anti-mouse ICAM-1 (1:10,000, R&D Systems). Both membranes were incubated with rabbit anti-glutaraldehyde-3-phosphate dehydrogenase (GAPDH, 1:1,000, Santa Cruz, CA, USA) as reference protein. Antibody binding was detected by incubation with appropriate peroxidase-conjugated secondary antibodies [all 1:2,000 except of anti-goat horseradish peroxidase (1:10,000), all Dako, Denmark] in blocking solution at room temperature for 1 h; for detection of ICAM-1 the Vectastain ABC-HRP Kit (Vector Laboratories, USA) was used according to the manufacturers’ instructions. Antibody binding was revealed by chemiluminescence using the enhanced chemiluminescence prime western blotting detection reagent (Amersham, GE Healthcare Life Science, USA). For visualization and densitometric analysis, the ChemiDocXRS+ imaging system and ImageLab software (Bio-Rad, Germany) were used.

### Processing of Peripheral Blood and Brain Tissues for Flow Cytometry

Isolation of single cell suspension for flow cytometry analysis was performed as previously described (28, 29). Briefly, animals were euthanized by i.p. injections of chloralhydrate (200 mg/kg body weight) followed by transcardial perfusion with ice-cold PBS and removal of brains. Brains were dissected and hemispheres divided into ipsilesional and contralesional parts. Two hemispheres were pooled per sample and homogenized through a 70-µm cell strainer (BD Biosciences) by continuous rinsing with 25 ml of cold HEPES-buffered RPMI1640. Samples were centrifuged at 400 × *g* for 10 min at 18°C. The supernatants were discarded and the pellets were resuspended in 15 ml of 37% Percoll in 0.01 N HCl/PBS and centrifuged at 2,800 × *g* for 20 min. Myelin was removed and the remaining cell pellet was washed twice in PBS.

Blood specimens were collected with ethylenediaminetetraacetate (EDTA) coated capillaries (CLINITUBES, Radiometer, Denmark) by snipping the right atrium of the heart immediately prior to intracardiac perfusion *via* the left ventricle. Blood samples were transferred into (EDTA) coated collection tubes (Minicollect, Greiner Bio One, Germany) and stored until further processing for a maximum of 30 min. Erythrocytes were lysed by incubation with lysis buffer (155 mM NH_4_Cl, 10 mM KHCO_3_, 3 mM EDTA) for 5 min followed by two washing steps with PBS.

### Flow Cytometry

Isolated cells were incubated with antibody cocktails for surface staining provided in Table S1 in Supplementary Material for 30 min at 4°C. For analysis of regulatory T cells, surface staining was followed by fixation and permeabilization with Fix/Perm buffer provided with the regulatory T cell staining kit (eBioscience, Germany) according to the manufactures’ instructions followed by intracellular staining of Foxp3 for 30 min at 4°C. Leukocyte subsets were identified and differentiated by their antigen expression using multichannel flow cytometry according to our previously established antibody panels (28). Due to inevitable cell loss by tissue processing (e.g., cytotoxic Percoll density gradient centrifugation) and low blood volumes accessible from neonatal mice, separate experiments for analysis of lymphoid cells (panel 1), regulatory T cells (panel 2), and myeloid (panel 3) cells were performed. Viable leukocytes were identified by gating for CD45 positive cells and FVD (fixable viability dye, eBioscience) negative cells. Microglia expressing low to intermediate levels of CD45 were excluded by gating on CD45^high^ cells (30, 31). Identified leukocytes were further divided into lymphoid subsets (panel 1), i.e., B cells (CD19), natural killer cells (NK1.1), CD4 and CD8 T cells (CD19^−^, NK1.1^−^ CD3^+^, CD4/8^+^). Regulatory T cells (panel 2) were identified as CD45^+/high^, CD3^+^, CD4^+^, Foxp3^+^ cells (Figures S1A,C in Supplementary Material). For analysis of depletion efficiency in anti-CD3-treated animals, CD3 was excluded from gating. With panel 3 (Table S1 in Supplementary Material), we distinguished neutrophils (lymphocyte^−^, Ly6G^+^), monocytes (lymphocyte^−^, CD115^+^), macrophages (lymphocyte^−^, Ly6G^−^, CD115^−^, CD11b^+^), and dendritic cells (lymphocyte^−^, Ly6G^−^, CD115^−^, CD11c^high^) (Figure S1B in Supplementary Material). Resident and inflammatory monocyte/macrophage subsets were distinguished according to their Ly6C expression. Data acquisition and analysis were performed on a BD FACS LSRII equipped with FACS Diva software (BD Biosciences). Total cell counts were determined using BD TrueCount beads (BD) as previously described (29, 32). Inter-experimental variations due to isolation procedures were corrected by relating values of ipsilateral hemispheres to values of contralateral hemispheres of the same animals.

### Statistical Analysis

All results were expressed as box and whisker plots with median values. Whiskers display the highest and the lowest value of the total data set. For statistical analysis, the GraphPad Prism 6.0 software package (GraphPad Software) was used. Data were tested for Gaussian distribution with the D’Agostino and Pearson omnibus normality test and then analyzed either by ordinal one-way ANOVA or by Kruskal–Wallis (non-parametric) with *post hoc* Bonferroni correction for multiple comparisons or with Dunn multiple comparison tests, respectively. When two groups were compared, unpaired, two-tailed Student’s *t-*test or Mann–Whitney test (non-parametric) were applied. In all analyses, *p* < 0.05 was considered statistically significant.

## Results

### FTY720 Induces Sustained T Cell Depletion in Neonatal Mice

**Figure 1:**
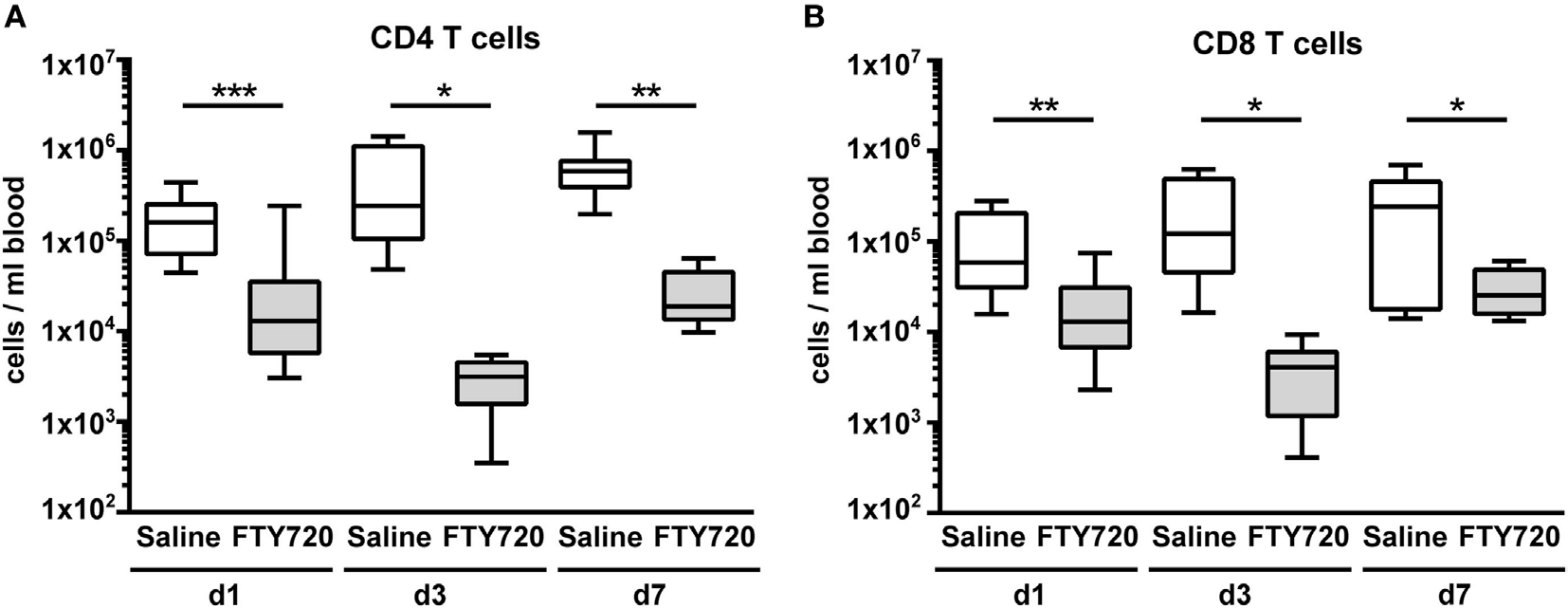
A single injection of FTY720 in postnatal day 9 mice results in a strong and sustained depletion of circulating T cells. Naïve 9-day-old C57BL/6 mice received a single intraperitoneal injection of 1 mg/kg FTY720 (in 0.9% NaCl). Saline-treated animals served as control. One, 3, and 7 days after injection, CD4 T cells **(A)**, CD8 T cells **(B)** were quantified in the blood *via* flow cytometry. Blood samples were collected from the right atrium of the heart and transferred into ethylenediaminetetraacetate coated collection tubes followed by erythrocyte lysis. Cell-specific antigens were stained with appropriate antibodies and viable leukocytes were identified by gating for CD45^+^ cells and FVD^−^ and further divided into CD4 and CD8 T cells (CD19^−^, NK1.1^−^ CD3^+^, CD4/8^+^). FTY720 induced a strong and long-lasting reduction in the amount of peripheral CD4 and CD8 T cells by 80–99% compared to saline-treated mice. *n* = 11–13 for day 1, *n* = 8–10/group for day 3, *n* = 7–8/group for day 7, *p < 0.05, **p < 0.01, ***p < 0.001, Mann–Whitney test for day 1, unpaired *t*-test for day 3 and day 7.

From adult brain injury models it is suggested that FTY720 mainly acts by sequestering circulating lymphocytes in lymphoid organs through internalization of S1P receptors resulting in reduced amounts of circulating and thus of CNS-infiltrated cells (14, 15, 20). Therefore, we first assessed the number of different lymphocyte subsets *via* flow cytometry 1, 3, and 7 days after a single injection of vehicle (saline) or FTY720 (1 mg/kg body weight i.p.) to 9-day-old naïve C57BL/6 mice. FTY720 induced a strong and long-lasting reduction in the amount of peripheral CD4 and CD8 T cells by 80–99% compared to saline-treated mice (Figure 1). The amount of B and natural killer cells was not significantly modulated by FTY720 treatment (Figure S2 in Supplementary Material).

### Peripheral T Cell Depletion by FTY720 Exacerbates Neonatal Hypoxic-Ischemic Brain Injury

**Figure 2:**
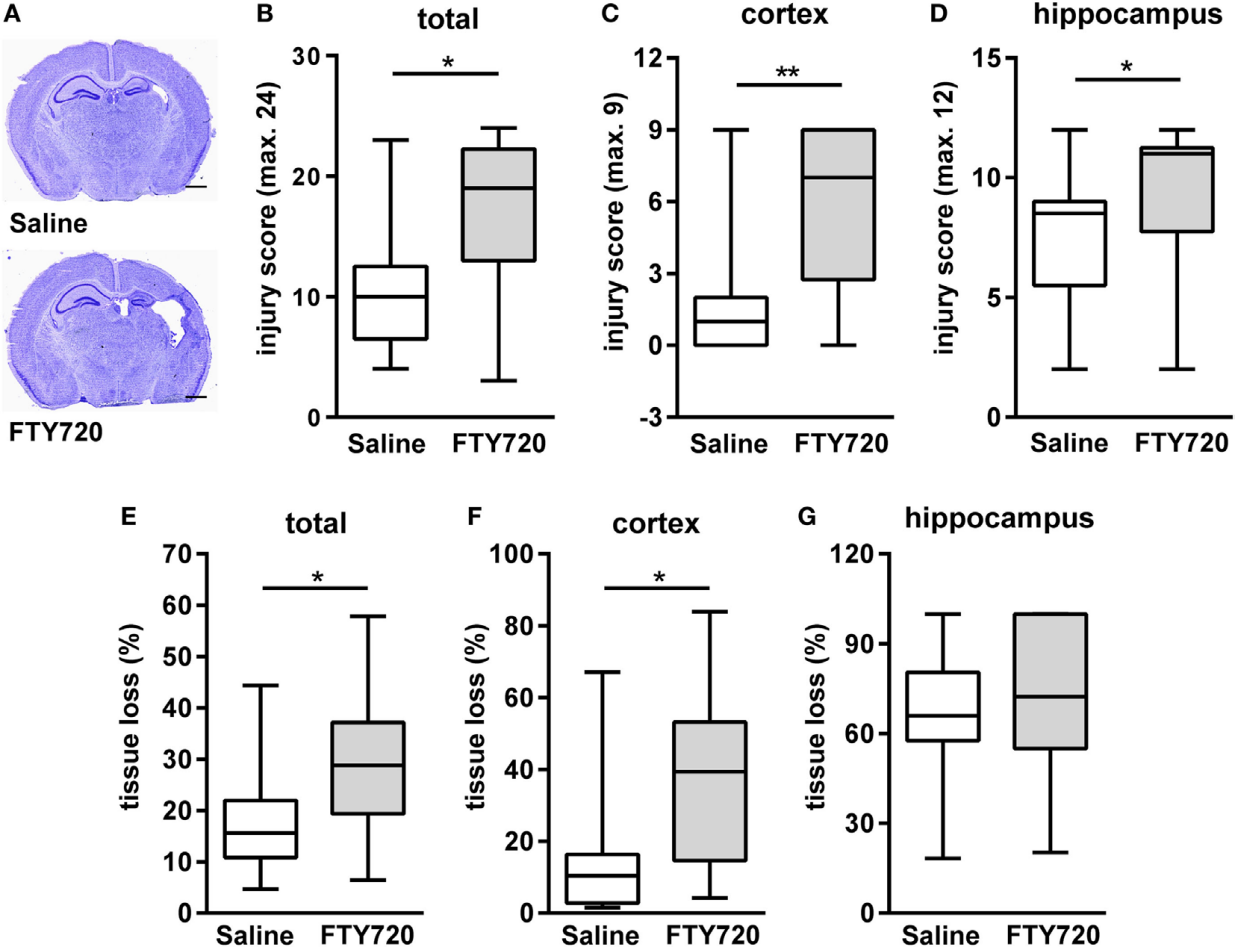
FTY720 treatment exacerbates brain injury 1 week after neonatal HI. Histological brain injury was determined on cresyl violet stained 20 µm cryostat sections of P16 mice that were exposed to HI followed by a single intraperitoneal injection of 1 mg/kg FTY720 or 0.9% NaCl (saline) on P9. **(A)** Representative images of brain sections (−1.9 to −2.0 mm from bregma) for each experimental group are shown (scale bar: 1 mm). **(B)** Injury scores were assessed in different brain regions, e.g., cortex **(C)** and hippocampus **(D)** according to a previously described scoring system in eight regions. Each region was given a rating from 0 to 3 (0—no detectable cell loss, 1—small focal areas of neuronal cell loss, 2—columnar damage in the cortex or moderate to severe cell loss in the other regions, 3—cystic infarction and gliosis). The sum score from different regions (cortex, hippocampus, and striatum) was calculated for each animal resulting in a total maximum score of 24. **(E–G)** Brain atrophy was assessed at the level of the striatum (+0.2 to +0.3 mm from bregma) and hippocampus (−1.9 to −2.0 mm from bregma) in two consecutive sections per region and animal by measurement of intact areas in ipsilateral and contralateral hemispheres using Image J. Tissue loss was determined by comparison with contralateral values. Overall and local cortical tissue injury was significantly increased by FTY720 treatment. *n* = 14–16/group, *p < 0.05, **p < 0.01, Mann–Whitney test.

Because of previous reports on the detrimental role of peripheral T cells in neonatal and adult ischemic brain injury (12, 26, 33, 34) we hypothesized that FTY720-induced depletion of circulating T cells ameliorates HI-induced brain injury. Therefore, we analyzed histopathological changes and brain atrophy in cresyl violet stained brain sections 1 week post HI (Figure 2A). Neuropathological assessment revealed that overall and local cortical and hippocampal tissue injury was significantly increased by FTY720 treatment (Figures 2B–D). In addition, total and cortical HI-induced brain tissue loss was significantly enhanced in FTY720-treated animals compared to saline-treated mice (Figures 2E,F). No significant differences were determined for hippocampal atrophy (Figure 2G). In the striatum no differences were observed for neuropathology and tissue atrophy (Figure S3 in Supplementary Material), confirming regional vulnerability to HI injury (35, 36) and regional variability to exogenous interventions (23, 37).

**Figure 3:**
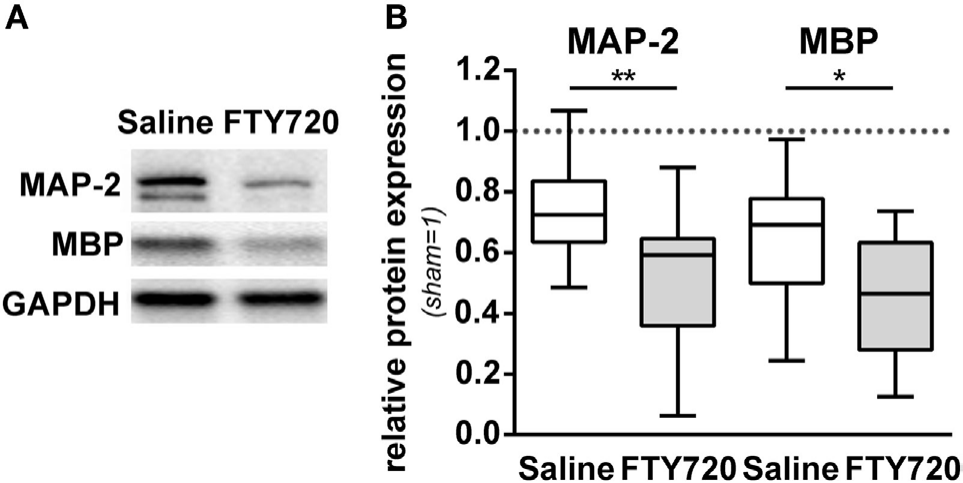
HI-induced gray and white matter loss is increased by FTY720 treatment. P9 mice were exposed to HI followed by a single intraperitoneal injection of 1 mg/kg FTY720 or 0.9% NaCl (saline). One week after HI, protein lysates were prepared from ipsilateral hemispheres at the hippocampal level (−2.0 to −2.3 mm from bregma) from brain sections of 200 µm thickness. Following gradient sodiumdodecylsulphate electrophoreses and protein transfer, membranes were incubated with anti-myelin basic protein (MBP), anti-microtubuli associated protein-2 (MAP-2), and anti-glutaraldehyde-3-phosphate dehydrogenase (GAPDH) antibodies **(A)**. For quantification data were normalized to the reference protein GAPDH and sham controls **(B)**. HI-induced loss of MAP-2 and MBP was significantly aggravated by FTY720 treatment. *n* = 14/group, *p < 0.05, **p < 0.01, unpaired *t*-test.

For further insight into target structures with respect to sub-acute gray and white matter injury, western blot analyses for MAP-2 and MBP expression at the level of the hippocampus were performed (Figure 3A). HI-induced loss of MAP-2 and MBP was significantly aggravated by FTY720 treatment (Figure 3B).

### FTY720 Does Not Alter Local Inflammatory Responses and Overall Leukocyte Infiltration

**Figure 4:**
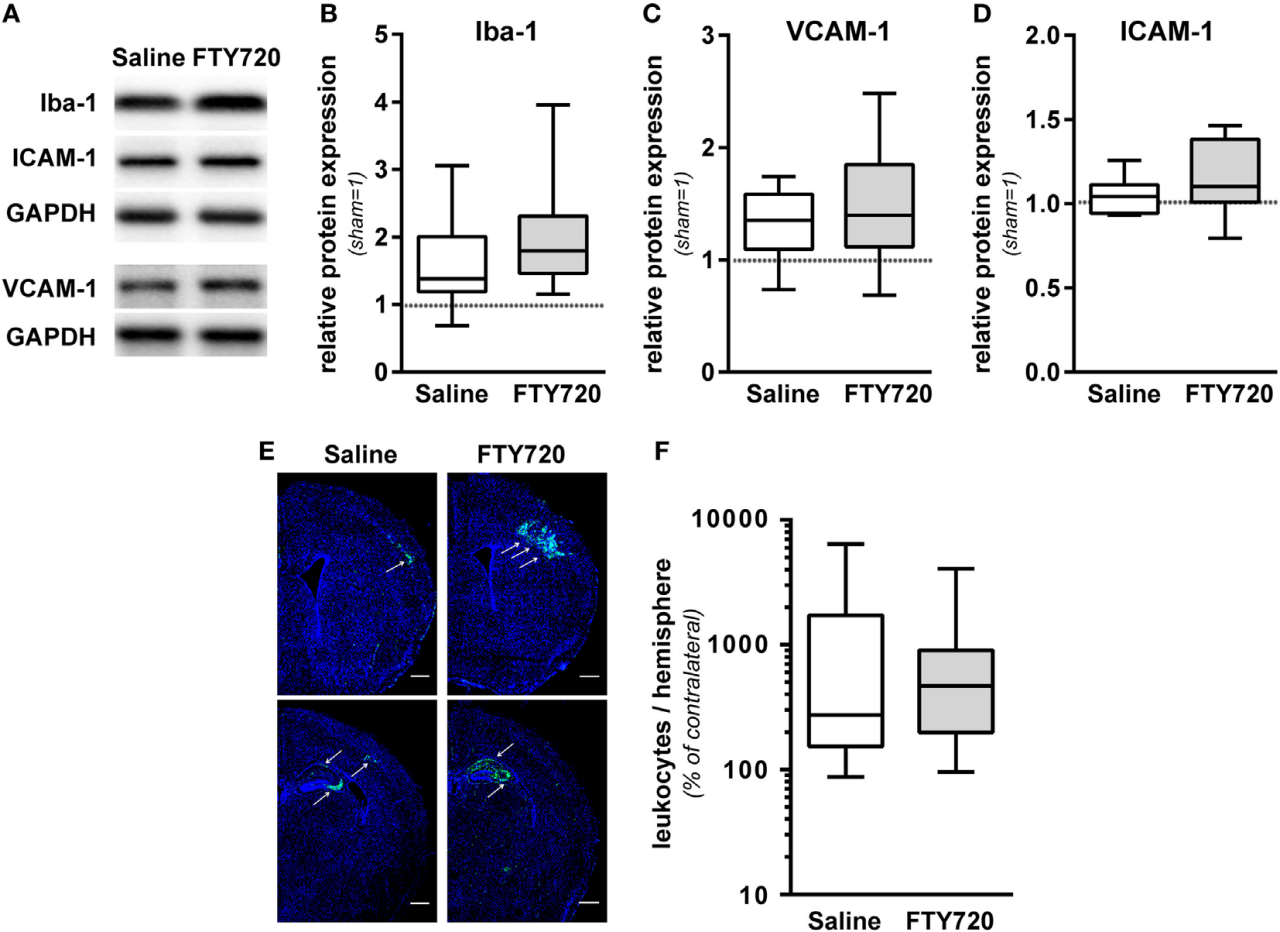
FTY720 does not alter microglia and endothelial activation and total leukocyte infiltration. P9 mice were exposed to HI followed by a single intraperitoneal injection of 1 mg/kg FTY720 or 0.9% NaCl (saline). **(A)** On P16 microglia and endothelial activation was assessed by quantification of Iba-1 **(B)**, VCAM-1 **(C)**, and ICAM-1 **(D)** in protein lysates *via* western blot. Protein lysates were prepared from ipsilateral hemispheres at the hippocampal level (−2.0 to −2.3 mm from bregma) from brain sections of 200 µm thickness followed by gradient sodiumdodecylsulphate (SDS) electrophoresis (Iba-1 and ICAM-1) or protein separation in 12.5% SDS gels. After protein transfer, membranes were incubated with appropriate antibodies. Data were normalized to the reference protein glutaraldehyde-3-phosphate dehydrogenase (GAPDH) and sham controls. FTY720 treatment did not significantly alter Iba-1, VCAM-1, and ICAM-1 protein levels. **(E)** Leukocyte infiltration was evaluated qualitatively by immunohistochemistry for CD45 (green) 1 week after HI. Nuclei were counterstained with Dapi (blue). Representative images show maximal intensity projections of confocal z-stacks at the level of striatum and at the level of the hippocampus (scale bar: 500 µm). Leukocyte infiltration was most prominent in regions mainly affected by the detrimental effect of FTY720, e.g., the hippocampus and cortex (indicated by arrows) **(E)**. Quantification of leukocyte infiltration was performed by flow cytometry after isolation of single cell suspensions and myelin removal *via* Percoll gradient centrifugation **(F)**. Absolute cell counts of viable CD45^high^ cells were determined in contralateral and ipsilateral hemispheres using TrueCount beads. Numbers in ipsilateral hemispheres were related to contralateral hemispheres of the same animals to correct for inter-experimental variations due to isolation procedures. Two hemispheres were pooled per sample. Total HI-induced peripheral leukocyte infiltration was not significantly changed by FTY720. *n* = 14/group for **(A–D)**, *n* = 30–31 samples/group [pooled analysis from measurements for panel 1/lymphoid, panel 2/regulatory T cells (Figure 5A), and panel 3/myeloid (Figure 5B)] for **(E)**.

To dissect the underlying mechanisms, we evaluated sub-acute neuroinflammatory responses including microglia and endothelial activation and peripheral leukocyte infiltration 1 week after HI. This time point was chosen to directly compare neuropathological findings with inflammatory responses since previous reports suggested a delayed infiltration of peripheral T cells into the neonatal hypoxic-ischemic brain (4–6, 11). FTY720 treatment induced a slight but not significant increase in microglia activation, assessed by Iba-1 protein expression (Figures 4A,B). Protein levels of the adhesion molecules VCAM-1 and ICAM-1, facilitating peripheral immune cell infiltration, were not significantly modulated by FTY720 (Figures 4A,C,D). In spite of profound and sustained lymphopenia in the peripheral blood (Figure 1), overall HI-induced peripheral leukocyte infiltration into HI-injured brains, qualitatively assessed by immunohistochemistry (Figure 4E) and quantified by flow cytometry (Figure 4F), was not significantly changed by FTY720 (Figures 4E,F). Interestingly, leukocyte infiltration was most prominent in regions mainly affected by the detrimental effect of FTY720, e.g., the hippocampus and cortex (Figures 4E and 2A,C,D), whereas less infiltration was observed in regions not affected by FTY720-induced exacerbation of brain injury, i.e., the striatum (Figure 4E; Figure S3 in Supplementary Material).

### Systemic T Cell Depletion by FTY720 Leads to Increased Infiltration of Innate Immune Cell Subsets Into the Neonatal Hypoxic-Ischemic Brain

**Figure 5:**
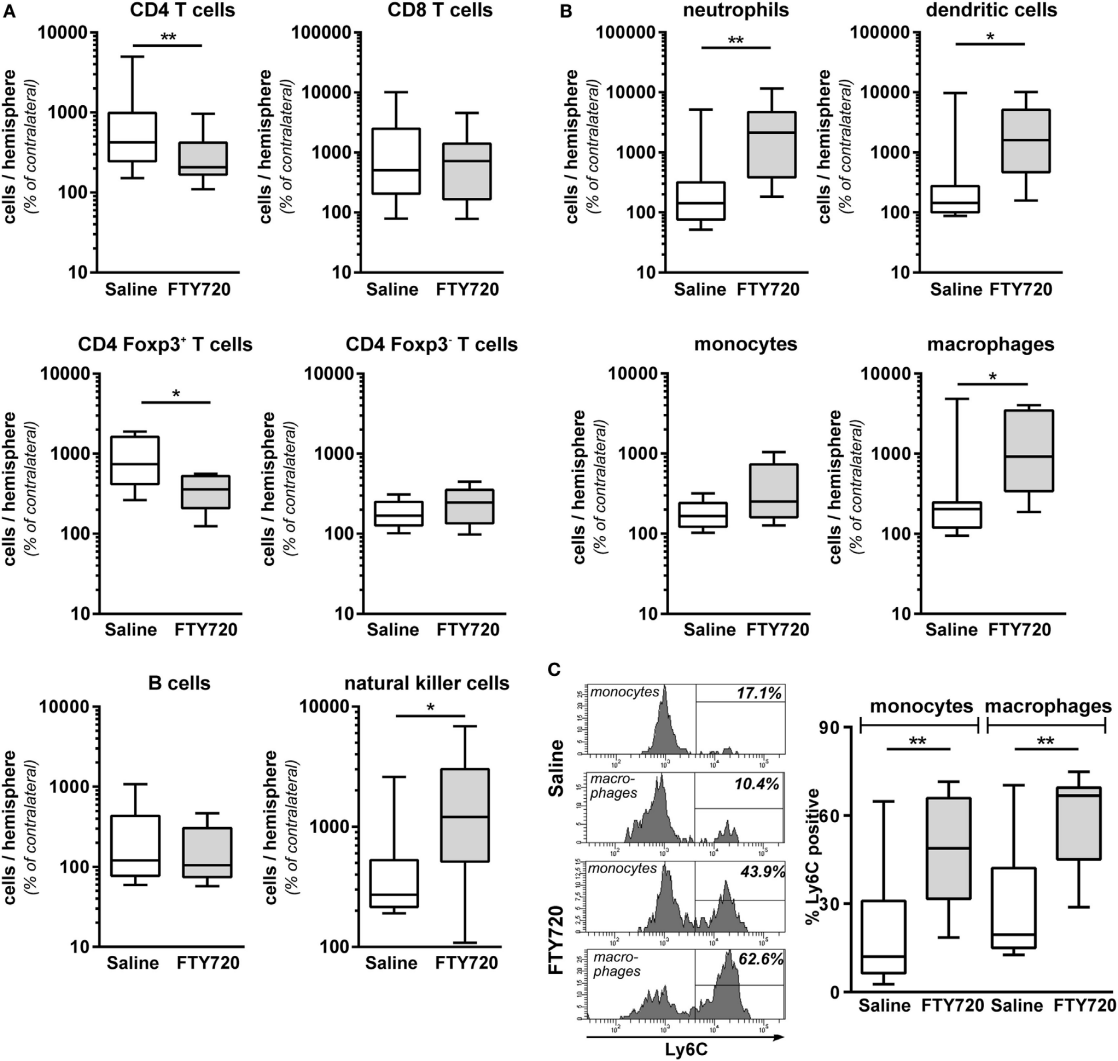
Peripheral T cell depletion by FT720 reduces the amount of T cells but increases innate immune cells in the hypoxic-ischemic brain. Multichannel flow cytometry was performed on immune cells isolated from ipsilateral and contralateral hemispheres obtained from P16 mice that were exposed to HI followed by a single intraperitoneal injection of 1 mg/kg FTY720 or 0.9% NaCl (saline) on P9. Different immune cell subsets were identified according to the gating strategy provided in Figure S1 in Supplementary Material. Briefly, viable peripheral leukocytes were identified as CD45^high^FVD (fixable viability dye)^−^ cells and further subdivided into B lymphocytes (CD19^+^), natural killer cells (NK1.1^+^), CD4 T cells (CD19^−^, NK1.1^−^, CD3^+^, CD4^+^), CD8 T cells (CD19^−^, NK1.1^−^, CD3^+^, CD8^+^), and regulatory T cells (CD3^+^, CD4^+^, Foxp3^+^) T cells **(A)**. For analysis of myeloid subsets viable leukocytes were gated for lymphocyte-depleted cells (CD3^−^, NK1.1^−^, B220^−^) and further subdivided into monocytes (CD115^+^, Ly6G^−^) and neutrophils (CD115^−^, Ly6G^+^). Remaining cells were gated by CD11b and CD11c to distinguish dendritic cells (Ly6G^−^, CD115^−^, CD11c^high^) and macrophages (Ly6G^−^, CD115^−^, CD11c^−^, CD11b^+^) **(B)**. Absolute lymphocyte **(A)** and myeloid **(B)** cell counts were quantified in contralateral and ipsilateral hemispheres. Numbers in ipsilateral hemispheres were related to contralateral hemispheres of the same animals to correct for inter-experimental variations due to isolation procedures. For monocytes and macrophages, the proportion of inflammatory cells was quantified based on their Ly6C expression **(C)**. FTY720 treatment significantly reduces the amount of infiltrated CD4 T cells and particularly regulatory T cells, which was accompanied by an increased infiltration of innate especially inflammatory myeloid cell types. *n* = 8–22 samples/group (two hemispheres pooled per sample), *p < 0.05, **p < 0.01, unpaired *t*-test for Foxp3^+^ and Foxp3^−^ CD4 T cells in **(A)** and monocytes in **(B)**, remaining: Mann–Whitney test.

Due to the surprising finding that peripheral T cell depletion by FTY720 results in similar infiltration of peripheral immune cells in general, we performed detailed analyses on the composition of the CNS infiltrate. We detected a significantly reduced amount of infiltrated CD4 T cells after HI in FTY720-treated animals compared to saline-treated mice (Figure 5A). CD4 T cell subset analysis revealed a significant reduction of Foxp3 positive regulatory T cells while infiltration of Foxp3 negative effector T cells was not changed in injured hemispheres of FTY720-treated animals (Figure 5A). These differences are caused by a selective infiltration of regulatory T cells after neonatal HI which is demonstrated by a 9.1-fold increase in regulatory T cells but only a 1.8-fold increase of effector T cells in ipsilateral hemispheres compared to contralateral parts in saline-treated control mice (Figure 5A).

Considering that the overall amount of infiltrated leukocytes was similar between both groups (Figures 4E,F), these results suggested an increased abundance of other immune cell subsets in the brain of FTY720-treated animals. Indeed, infiltrated cell numbers of different innate immune cell subsets, such as natural killer cells, neutrophils, dendritic cells, and macrophages (Figures 5A,B) were significantly increased in animals with FTY720-induced systemic T cell depletion compared to saline-treated animals. Considering the heterogeneity of monocyte/macrophage phenotypes, we further distinguished between resident and inflammatory cells based on their Ly6C expression (Figure 5C). The percentage of Ly6C^+^ inflammatory cells was significantly increased in the macrophage and monocyte population of T cell-depleted mice (Figure 5C). These results suggest that FTY720-induced depletion of T cells in the periphery and the hypoxic-ischemic brain is counteracted by an increased infiltration of innate and particularly inflammatory immune cell subtypes resulting in a similar amount of total leukocytes in the ipsilateral injured hemisphere in both experimental groups (Figures 4E,F). This was confirmed by analyzing the percentage of different immune cell subsets in the total leukocyte population in the brain. While FTY720 treatment resulted in a reduction of T cell frequency from 1.6 to 0.5% of total viable leukocytes, a significant increase in neutrophil and dendritic cell percentages from 11.1 to 29.6% (*p* < 0.05) and from 1.9 to 3.5% (*p* < 0.05), respectively, was detected in ipsilateral hemispheres.

**Figure 6:**
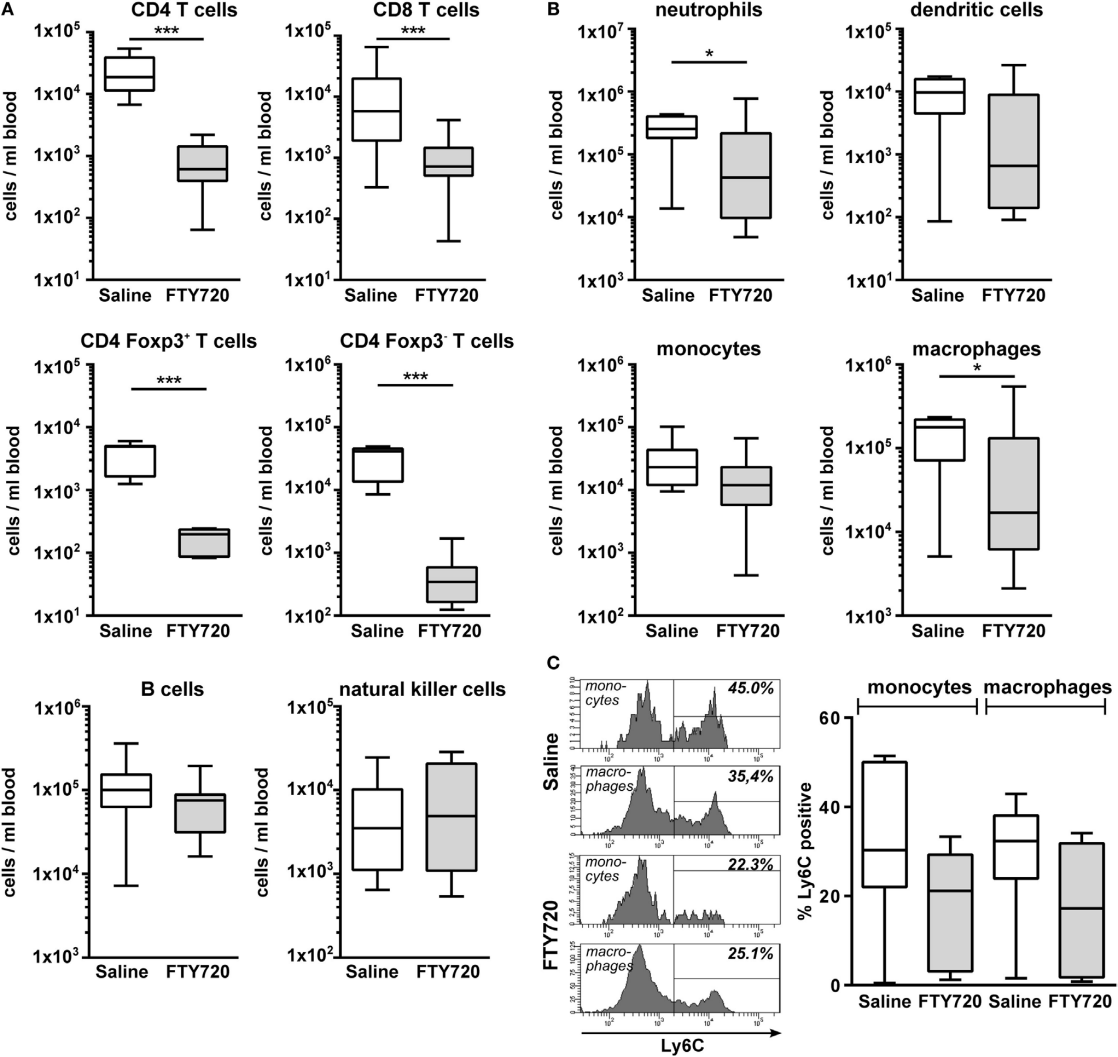
FTY720 treatment results in reduced peripheral T cell, neutrophil, and macrophage counts in neonatal HI-injured mice. Immune cell subset analysis in the peripheral blood was performed in P16 mice that were exposed to HI followed by a single intraperitoneal injection of 1 mg/kg FTY720 or 0.9% NaCl (saline) on P9. Different immune cell subsets were identified according to the gating strategy provided in Figure S1 in Supplementary Material. Briefly, viable peripheral leukocytes were identified as CD45^+^FVD (fixable viability dye)^−^ cells and further subdivided into B lymphocytes (CD19^+^), natural killer cells (NK1.1^+^), CD4 T cells (CD19^−^, NK1.1^−^, CD3^+^, CD4^+^), CD8 T cells (CD19^−^, NK1.1^−^, CD3^+^, CD8^+^), and regulatory T cells (CD3^+^, CD4^+^, Foxp3^+^) T cells **(A)**. For analysis of myeloid subsets viable leukocytes were gated for lymphocyte-depleted cells (CD3^−^, NK1.1^−^, B220^−^) and further subdivided into monocytes (CD115^+^, Ly6G^−^) and neutrophils (CD115^−^, Ly6G^+^). Remaining cells were gated by CD11b and CD11c to distinguish dendritic cells (Ly6G^−^, CD115^−^, CD11c^high^) and macrophages (Ly6G^−^, CD115^−^, CD11c^−^, CD11b^+^) **(B)**. Absolute lymphocyte **(A)** and myeloid **(B)** cell counts were quantified. For monocytes and macrophages, the proportion of inflammatory cells was quantified based on their Ly6C expression **(C)**. The amount of circulating T cells and of neutrophils and macrophages is significantly reduced in FTY720-treated HI mice, while the number of B, natural killer, and dendritic cells remained unchanged. *n* = 7–19/group unpaired *t*-test for Foxp3^+^ and Foxp3^−^CD4 T cells in **(A)** and Ly6C^+^ cells in **(C)**, remaining: Mann–Whitney test.

To clarify whether the increased amount of innate immune cells in the hypoxic-ischemic brain was caused by in increased transmigration or rather reflected changes in the periphery, we quantified immune cell subtypes in the blood 1 week after HI. Confirming our results obtained in naïve mice (Figure 1), we detected a significantly reduced amount CD4 and CD8 T cells in FTY720-treated HI mice while the number of B and natural killer cells remained unchanged (Figure 6A). Detailed analysis of CD4 T cells revealed that Foxp3 positive regulatory T cells were similarly reduced as Foxp3 negative effector T cells by 95.5 and 98.4%, respectively (Figure 6A). However, we determined a significantly lower proportion of circulating regulatory T cells in the total CD4 T cell population compared to the frequency of CNS-infiltrated regulatory T cells (11.2 vs. 55.6%, *p* < 0.001; Figures 5A and 6A) in saline-treated mice, confirming the selective infiltration of regulatory T cells after neonatal HI. Interestingly, the amount of circulating neutrophils and macrophages was significantly decreased in FTY720-treated mice contrasting findings in the brain (Figure 6B). Similarly, the proportion of inflammatory monocytes and macrophages was reduced, though not reaching significance (Figure 6C).

### Detrimental Impact of FTY720 on Neonatal HI-Induced Brain Injury Depends on Peripheral T Cell Depletion

**Figure 7:**
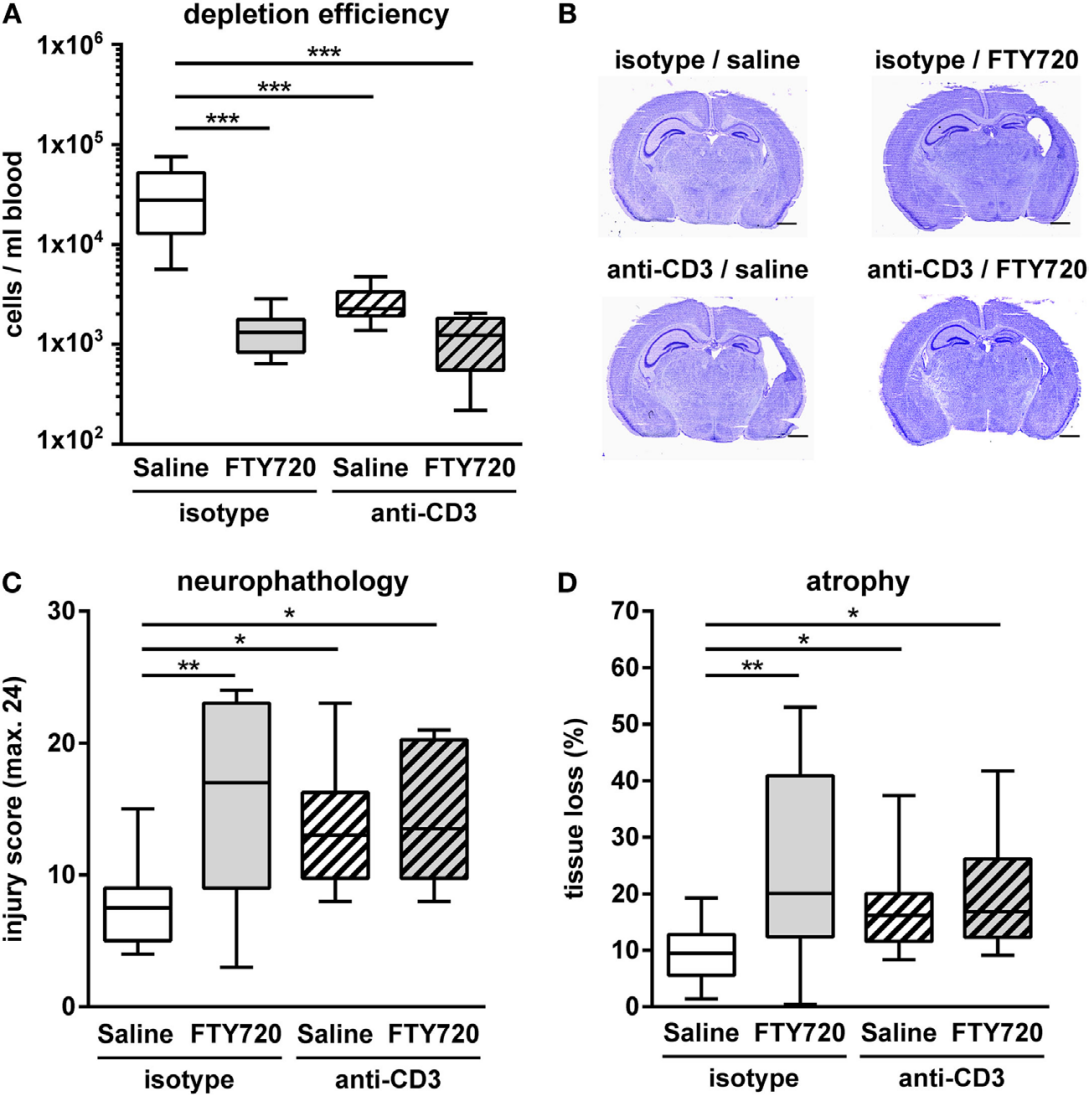
Exacerbation of HI-induced brain injury by FTY720 is dependent on peripheral T cells. Whether FTY720s’ detrimental effect could be attributed to its T cell depleting effect was determined in anti-CD3-treated animals. P9 mice were exposed to HI followed by a single intraperitoneal injection of 1 mg/kg FTY720 or 0.9% NaCl (saline). Anti-CD3 treatment was started on P8 followed by repetitive injections every 48 h according to our previous report (26). Control mice received an isotype control antibody. One week after HI, depletion efficiency by antibody- and FTY720-treatment as well as in the combined setting was analyzed in the peripheral blood *via* flow cytometry **(A)**. Brain injury was evaluated on cresyl violet stained tissue sections on P16 **(B,C)**. Neuropathological assessment was performed according to a previously described scoring system in eight regions. Each region was given a rating from 0 to 3 (0—no detectable cell loss, 1—small focal areas of neuronal cell loss, 2—columnar damage in the cortex or moderate to severe cell loss in the other regions, 3—cystic infarction and gliosis). The sum score from different regions (cortex, hippocampus, and striatum) was calculated for each animal resulting in a total maximum score of 24 **(C)**. Brain tissue atrophy was determined by measurement of intact areas in ipsilateral and contralateral hemispheres using Image J. Tissue loss was determined by comparison with contralateral values **(D)**. Anti-CD3 and FTY720-mediated T cell depletion significantly increased HI-induced injury. The combined treatment resulted in a similar degree of injury compared to single treatments. *n* = 8–10/group for **(A)**, *n* = 10–14/group for **(B–D)**, one-way ANOVA with Bonferroni *post hoc* test for **(A,C)**, Kruskal–Wallis with Dunn’s multiple comparison test for **(D)**.

Whether exacerbation of ischemic brain injury in neonatal mice could be attributed to FTY720s’ lymphopenic mode of action or is caused by direct neurotoxic effects was assessed in T cell depleted mice. T cell depletion was performed by anti-CD3 antibody treatment according to our previous report (26). Of note, the selected treatment protocol resulted in a strong reduction of peripheral T cells by 92% similar to that obtained after FTY720 treatment (Figure 7A). Neuropathological assessment and quantification of brain tissue loss revealed that antibody- and FTY720-mediated T cell depletion significantly increased HI-induced injury (Figures 7B–D). Of note, there was no further exacerbation of damage after FTY720 treatment in T cell-depleted mice (Figures 7B–D).

## Discussion

In contrast to adult stroke, peripheral T cell depletion by pharmacological and antibody-mediated intervention increases brain injury in a term-born equivalent model of hypoxic-ischemic brain injury. Analysis of peripheral and cerebral leukocyte subsets provided further insights into potential underlying mechanisms involving an increased infiltration of innate and particularly inflammatory cell types into the injured neonatal brain when T cells are lacking. Our unexpected results contrast findings in adult ischemia, but confirm previous reports about questionable translation from adults to neonates in experimental studies (38).

The concomitant increased infiltration of inflammatory cells, mainly neutrophils and inflammatory macrophages in the absence of T cells suggests that they have contributed to increased HI-induced brain injury. This is supported by previous work demonstrating that antibody-mediated depletion of circulating neutrophils reduces neonatal and adult ischemic brain injury (26, 39). Simultaneously reduced cell numbers of neutrophils and macrophages in the peripheral blood implicate a redistribution of inflammatory cells from the circulation to the hypoxic-ischemic brain with the total amount of infiltrated leukocytes being similar.

Expression of general endothelial adhesion molecules (i.e., VCAM and ICAM) was not modulated, suggesting that neonatal T cells regulate the migratory capacity of peripheral inflammatory cell types. So far, we can only speculate about the potential mechanisms. However, the described bias toward anti-inflammatory T cell subsets in neonates (40) might provide an endogenous protective mechanism of the neonatal organism exposed to an acute hypoxic-ischemic insult to limit excess inflammation. In the present work, we demonstrate a selective infiltration of regulatory T cells into the neonatal hypoxic-ischemic brain. Therefore, overall peripheral depletion of T cells, similarly affecting regulatory and effector T cells results in a more pronounced difference for cerebral regulatory T cell counts compared to effector T cells in HI-injured brain hemispheres. This suggests that in addition to inhibition of the migratory capacity of inflammatory cells in the periphery, the presence of regulatory T cells in the injured brain might be an important mechanism of endogenous neuroprotection. To develop optimal and specific therapies, further work is needed to specify the temporal regulation of this T cell subset and corresponding molecular mechanisms mediating neuroprotection.

In spite of these interesting findings, our results in part contradict to a previous report describing neither protection nor exacerbation of HI-induced brain injury after FTY720 treatment in postnatal day 7 rats (22). Differences in species, age, and injury severity likely provide an explanation. As such, P7 rats compared to P9 mice were used and control animals revealed up to 40% tissue loss in the cortex while in the present study on P9 mice only 10% cortical tissue loss was observed. Severity of injury and differences in experimental models are of particular importance regarding the neuroinflammatory response as already reported in adult models of ischemic stroke (41, 42). Furthermore, FTY720 was purchased from different companies potentially affecting its lymphopenic capacity. This can hardly be clarified as depletion efficiency and duration in terms of absolute T cell numbers and other leukocyte subsets were not provided (22).

To determine whether detrimental effects were caused by FTY720s’ pleiotropic effects (43) independent of its lymphopenic mode of action we performed T cell depletion *via* antibody treatment which resulted in increased brain injury comparable to that induced by FTY720 single treatment. The fact that FTY720 did not further enhance HI brain injury in T cell depleted mice suggests that side effects on other neural cell types are rather unlikely. This is supported by previous work with neuronal cell cultures demonstrating no effect of FTY720 on hypoxia-induced neuronal cell death (19, 20) and by results of the present study revealing no significant changes in microglia and endothelial activation. However, we cannot completely exclude the possibility that FTY720 might have directly affected innate immune cells as previously suggested in an experimental model of adult stroke (44).

Our results of aggravated brain injury after peripheral T cell depletion seem contradicting to previous reports in neonatal rodents showing neuroprotection in Rag1^−/−^ mice or by depletion of gamma delta T cells (11, 12). However, Rag1^−/−^ mice lack mature T but also B cells impeding clear conclusions about the specific contribution of each immune cell subset (11). Albertsson et al. specifically deleted gamma delta T cells resulting in significant neuroprotection after neonatal HI (12). Since the proportion of this subset is rather small within the total T cell population our results suggest that the detrimental role of gamma delta T cells might have been counteracted by other more abundant protective T cell subsets in the injured brain, e.g., regulatory T cells. This is supported by our results on a selective infiltration of regulatory T cells compared to Foxp3 negative effector T cells in HI-injured brain hemispheres.

In addition to the aforementioned explanations, the most important issue to be considered when comparing these two studies is the difference in the age of animals. While Nazmi and Albertsson investigated pre-term equivalent HI models using P4 and P5 mice we used a term-born equivalent injury model. This may strongly modulate the impact of immunomodulatory interventions because the immune system and the brain are still developing. As such, white matter development and axonal outgrowth in the rodent CNS between P1 and P7 correspond to 23–36 weeks gestation in humans (45). Therefore, rodents at P9–10 are meanwhile considered to be more comparable to term infants regarding brain development (46, 47). According to that, infiltrated T cells might interfere with vulnerable CNS maturation processes taking place between p4–p7 (48), while in the term-born equivalent brain of P9–P10 rodents T cells may rather protect from HI-induced destruction of already differentiated and matured neural cells, a hypothesis that needs to be proven in future studies. In addition, changes in the immune system between P5 and P9 might explain differences in outcome. Even though comprehensive ontogenetic investigations of different immune cell subsets in neonatal mice are sparse, few reports described pronounced changes in the immune system. These involve, for example, a continuous increase in the proportion of lymphocytes while neutrophils decrease within the first 2 weeks of life (49, 50). A detailed characterization of T cell phenotypes over time is still missing.

Instead of Rag1^−/−^ mice, we used an antibody-mediated cell depletion approach, which might not be as efficient as genetic ablation. However, a strong reduction of circulating T cells by 92% was achieved in the present work. To dissect the specific role of T cells in Rag1^−/−^ mice adoptive transfer of B cells is needed, which is technically challenging in neonatal mice *via* the intravenous route. Studies in adult brain ischemia revealed similar outcomes with regard to histological brain injury in Rag1^−/−^ reconstituted with B cells compared to anti-CD3 treatment (26, 33) suggesting that antibody-mediated depletion is an appropriate approach. In order to mimic FTY720-induced reduction in peripheral T cells, we performed depletion throughout the entire observation period. Considering the suggested biphasic T cell infiltration pattern (7), we cannot determine the relevance of these two infiltration peaks separately. More mechanistic analyses with time-specific deletion will be needed to clarify the pathophysiological role of T cell infiltration at early and later time points.

In conclusion, the present work demonstrates that in contrast to adult stroke. FTY720 worsens neonatal HI-induced brain injury, most likely through sustained depletion of peripheral T cells. These results highlight that caution is needed when transferring findings from adult animal models to the developing brain. The time point of injury and intervention seems to be critical for injury outcome after immunomodulatory interventions. Furthermore, the current results suggest that neonatal T cells promote endogenous neuroprotection with regulatory T cells of particular importance in this context. Further in depth analysis specifically targeting this T cell subset may provide new opportunities for therapeutic interventions.

## Ethics Statement

Experiments were performed in accordance to the Animal Research: Reporting of *In Vivo* Experiments (ARRIVE) guidelines with government approval by the State Agency for Nature, Environment and Consumer Protection North Rhine-Westphalia.

## Author Contributions

JH, CK, HA, MC, and IB performed experiments and analyzed the data. JH and IB initiated, designed, and organized the study. JH, IB, WH, and UF-M wrote the manuscript.

## Conflict of Interest Statement

The authors declare that the research was conducted in the absence of any commercial or financial relationships that could be construed as a potential conflict of interest.
