# Supplementary material for: Peripheral T Cell Depletion by FTY720 Exacerbates Hypoxic-Ischemic Brain Injury in Neonatal Mice
Source: Front Immunol. 2018 Aug 6;9:1696. doi: 10.3389/fimmu.2018.01696 (PMC6087766; doi:10.3389/fimmu.2018.01696)
Supplement: Supplementary file 1 [file data_sheet_1.pdf]

Supplementary Table and Figures

Josephine Herz[1]*, Christian Köster[1], Marius Crasmöller[1], Hanna Abberger[2], Wiebke Hansen[2], Ursula Felderhoff-Müser[1], Ivo Bendix[1]*

[1]*Department of Pediatrics 1, Neonatology and Experimental Perinatal Neuroscience, University Hospital Essen, University of Duisburg-Essen, Essen, Germany*

[2]*Institute of Medical Microbiology, University Hospital Essen, University of Duisburg-Essen, Essen, Germany*

Correspondence:

Josephine Herz

Ivo Bendix

Department of Pediatrics 1, Neonatology and Experimental Perinatal Neurosciences, University Hospital Essen, University of Duisburg-Essen, Essen, Germany

Hufelandstr. 55, 45147 Essen, Essen, Germany,

Phone: + 49 201 723-85187, + 49 201 723-2114

Fax: +49 201 723-5727

josephine.herz@uk-essen.de; ivo.bendix@uk-essen.de

Supplementary Table 1: Antibodies used for flow cytometry

antigen	conjugate	host / istoype	clone	supplier
**Panel 1**				
CD45	Alexa Fluor700	Rat / IgG2b, kappa	30-F11	BD Biosciences
CD3	Fitc	Rat / IgG2b, kappa	17A2	eBioscience
CD4	PE-Cy7	Rat / IgG2a, kappa	RM4-5	BD Biosciences
CD8a	PerCP	Rat / IgG2a, kappa	53-6.7	BD Biosciences
CD19	APC	Rat / IgG2a, kappa	eBio1D3	eBioscience
NK1.1	BD Horizon V450	Mouse / IgG2a, kappa	PK136	BD Biosciences
**Panel 2**				
CD45	Alexa Fluor700	Rat / IgG2b, kappa	30-F11	BD Biosciences
CD3	Fitc	Rat / IgG2b, kappa	17A2	eBioscience
CD8a	PerCP	Rat / IgG2a, kappa	53-6.7	BD Biosciences
CD4	Pacific Blue	Rat / IgG2a, kappa	RM4-5	BD
Foxp3	PE	Rat / IgG2a, kappa	FJK-16s	eBioscience
**Panel 3**				
CD45	Alexa Fluor700	Rat / IgG2b, kappa	30-F11	BD Biosciences
Ly6G	FITC	Rat / IgG2a, kappa	1A8	BD Biosciences
CD115	APC	Rat / IgG2a, kappa	AFS98	eBioscience
Ly6C	BD Horizon V450	Rat / IgM, kappa	AL-21	BD Biosciences
CD11b	PE-Cy7	Rat / IgG2b, kappa	M1/70	eBioscience
CD11c	PE	Hamster / IgG	N418	Miltenyi Biotec
CD3	PerCP-Cy5.5	American Hamster / IgG1, kappa	145-2C11	BD Biosciences
CD45R (B220)	PerCP-Cy5.5	Rat / IgG2a, kappa	RA3-6B2	eBioscience
NK1.1	PerCP-Cy5.5	Mouse / IgG2a, kappa	PK136	eBioscience

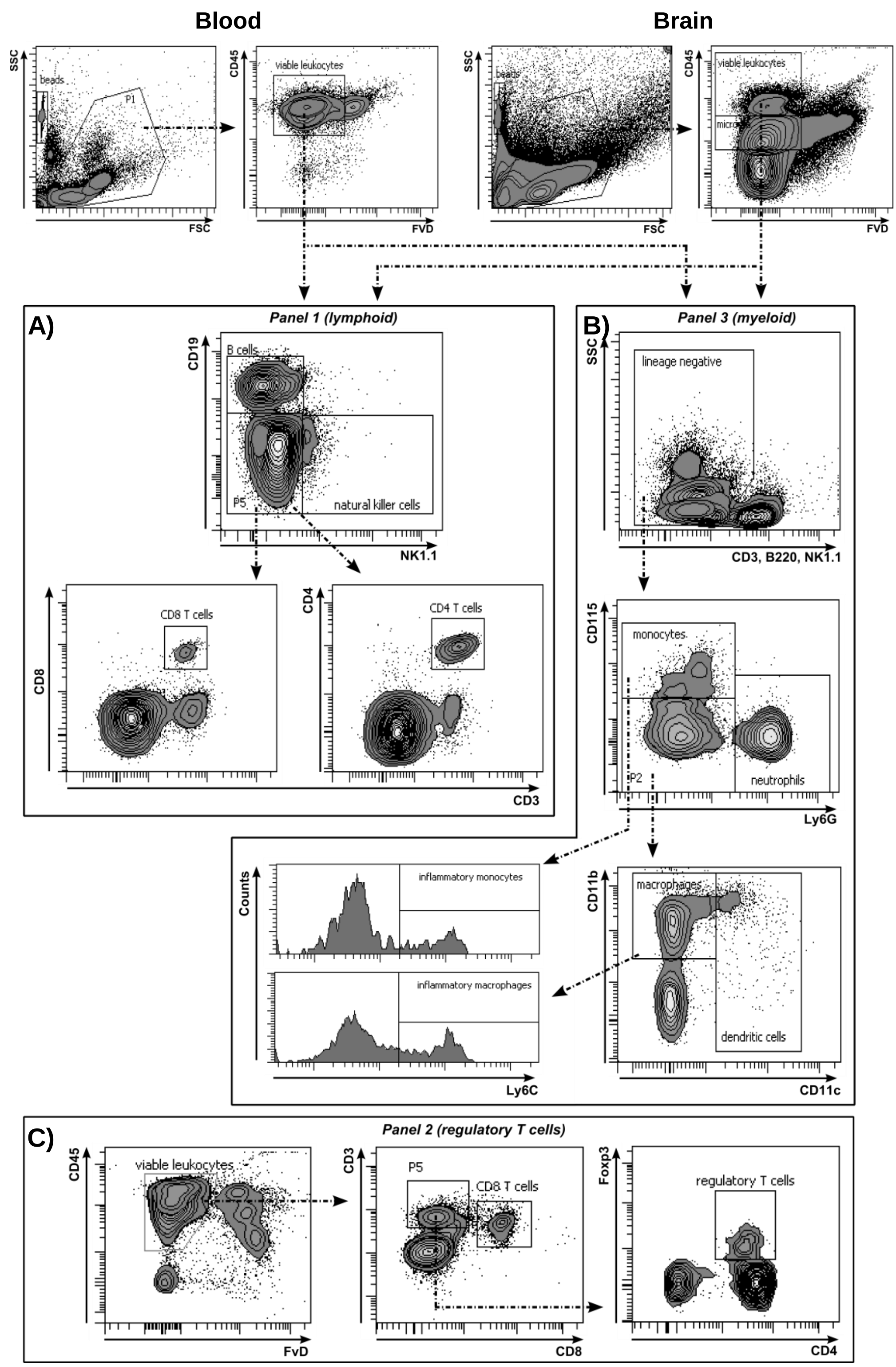

Supplementary Fig. S1: Gating strategy to identify leukocyte subsets by flow cytometry in blood and brain samples. Leukocytes were identified by their CD45 expression. For brain samples peripheral leukocytes were distinguished from microglia based on their high CD45 expression compared to low/intermediate expression of CD45 on microglia (Sedgwick et al., 1991;Mausberg et al., 2009;Ritzel et al., 2015). Viable cells were identified as FVD (fixable viability dye) negative cells. **(A)** For analysis of lymphoid cells (Supplementary Table 1,

Panel 1) $CD45^{+/high}FVD^{-}$ cells were gated for B lymphocytes (CD19) and natural killer cells (NK1.1). The remaining cells (P5) were gated for CD4 ($CD3^{+}CD4^{+}$) and CD8 ($CD3^{+}CD8^{+}$) T cells. **(B)** For myeloid subsets (Supplementary Table 1, Panel 3) viable leukocytes were gated for lymphocyte-depleted cells ($CD3^{-}$, $NK1.1^{-}$, $B220^{-}$) to exclude contamination with lymphoid subsets, which can partially express myeloid markers such as CD11b. This population was further subdivided by CD115 and Ly6G expression to identify monocytes and neutrophils, respectively. The remaining cells (P2) were gated by CD11b and CD11c to distinguish dendritic cells (Ly6G-, CD115-, $CD11c^{high}$) and macrophages ($Ly6G^{-}$, $CD115^{-}$, $CD11c^{-}$, $CD11b^{+}$). Inflammatory monocytes and macrophages were identified as $Ly6C^{+}$ cells. **(C)** Regulatory T cells (Supplementary Table 1, Panel 2) were identified within the $CD45^{+/high}CD3^{+}CD4^{+}$ population based on their Foxp3 expression.

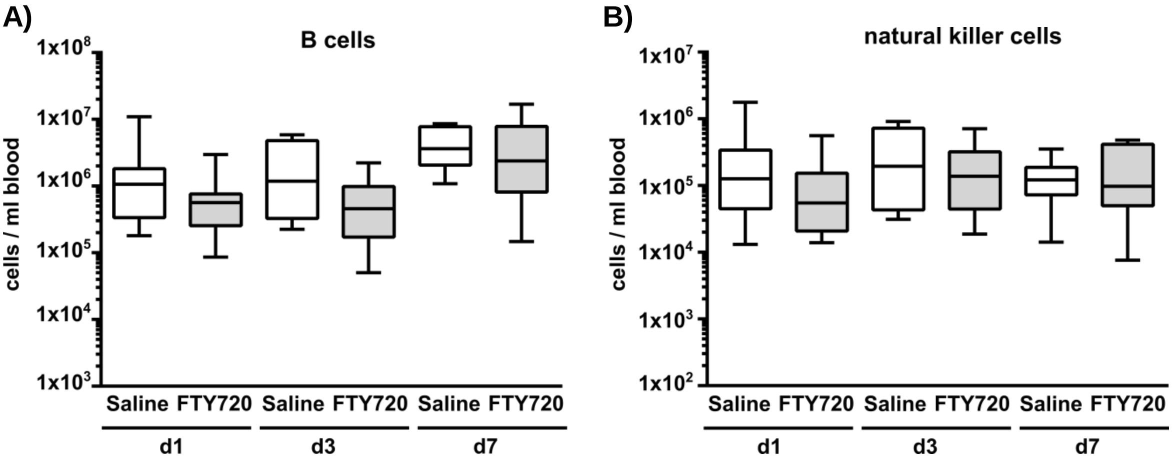

Supplementary Fig. S2: A single injection of FTY720 in postnatal day 9 mice does not modulate circulating B lymphocyte and natural killer cell counts. Naïve nine day old C57BL/6 mice received a single i.p. injection of 1 mg/kg FTY720 (in 0.9% NaCl). Saline-treated animals served as control. One, 3 and 7 days after injection, B lymphocytes ($CD45^+$, $CD19^+$) **(A)** and natural killer cells ($CD45^+NK1.1^+$) **(B)** were quantified in the blood via flow cytometry. n=11-13 for d1, n=8-10/group for d3, n=7-8/group for d7

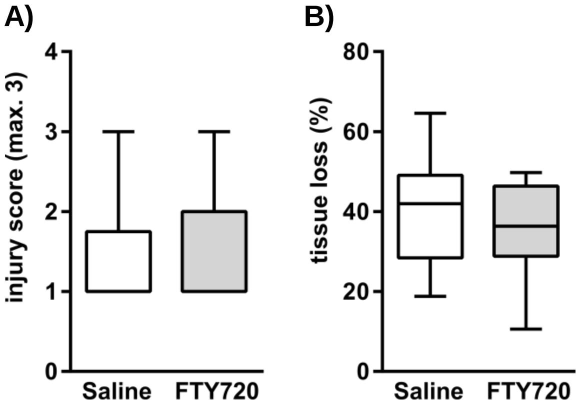

Supplementary Fig. S3: No impact of FTY720 treatment on HI-induced brain injury in the striatum. Histological brain injury was determined on cresyl violet stained 20 µm cryostat sections of P16 mice that were exposed to HI followed by a single i.p. injection of 1 mg/kg FTY720 or 0.9% NaCl (saline) on P9. **(A)** Injury scores were assessed in the striatum according to a previously described scoring system (Sheldon et al., 1998;Reinboth et al., 2016) with rating from 0 to 3 (0- no detectable cell loss, 1- small focal areas of neuronal cell loss, 2- moderate to severe cell loss, 3- cystic infarction and gliosis). **(B)** Striatal atrophy was assessed in two consecutive sections (+0.2 to +0.3 mm from bregma) through area measurement of demarcating striatal areas using Image J. n=14-16/group

Mausberg, A.K., Jander, S., and Reichmann, G. (2009). Intracerebral granulocyte-macrophage colony-stimulating factor induces functionally competent dendritic cells in the mouse brain. *Glia* 57**,** 1341-1350.

Reinboth, B.S., Koster, C., Abberger, H., Prager, S., Bendix, I., Felderhoff-Muser, U., and Herz, J. (2016). Endogenous hypothermic response to hypoxia reduces brain injury: Implications for modeling hypoxic-ischemic encephalopathy and therapeutic hypothermia in neonatal mice. *Exp Neurol* 283**,** 264-275.

Ritzel, R.M., Patel, A.R., Grenier, J.M., Crapser, J., Verma, R., Jellison, E.R., and Mccullough, L.D. (2015). Functional differences between microglia and monocytes after ischemic stroke. *J Neuroinflammation* 12**,** 106.

Sedgwick, J.D., Schwender, S., Imrich, H., Dorries, R., Butcher, G.W., and Ter Meulen, V. (1991). Isolation and direct characterization of resident microglial cells from the normal and inflamed central nervous system. *Proc Natl Acad Sci U S A* 88**,** 7438-7442.

Sheldon, R.A., Sedik, C., and Ferriero, D.M. (1998). Strain-related brain injury in neonatal mice subjected to hypoxia-ischemia. *Brain Res* 810**,** 114-122.
